# A register-based study: cough - a frequent phenomenon in the adult population

**DOI:** 10.1186/s12890-022-02228-z

**Published:** 2022-11-19

**Authors:** Vibeke Backer, Andreas Porsborg, Victor Hansen, Tina Skjold, Johannes Martin Schmid, Mette Kehlet, Christian Torp-Pedersen, Kristian Aasbjerg

**Affiliations:** 1 Department of otorhinolaryngology Head & Neck surgery, Rigshospitalet, Copenhagen University, Blegdamsvej 9, DK-2100 Copenhagen, Denmark; 2 Center of physical activity Research (CFAS), Rigshospitalet, Copenhagen University, Copenhagen, Denmark; 3 Department of Respiratory Medicine and Allergy, Aarhus University hospital, Aarhus University, Aarhus, Denmark; 4 MSD Denmark, Havneholmen 25, V, 1561 Copenhagen, Denmark; 5 Department of Cardiology, Nordsjaellands Hospital, Hillerød, Denmark; 6 Department of Cardiology, Aalborg University Hospital, Aalborg, Denmark; 7 Department of Public Health, University of Copenhagen, Copenhagen, Denmark

**Keywords:** Cough, Unexplained chronic cough, Refractory chronic cough, Register-based study, Nationwide cohort

## Abstract

**Background:**

Chronic cough, more than 8 weeks, can either be without co-morbidity called unexplained chronic cough (UCC) or with co-morbidity called refractory chronic cough (RCC). Using datasets from the Danish National Prescription Registry (Prescription Registry) and Danish National Patient Registry (Patient Registry) we wanted to investigate the prevalence and factors of importance of cough in a Nationwide registry.

**Material and methods:**

Inclusion criteria were patients 18–90 years with at least one final cough diagnosis (ICD-10 DR05/DR059) in Patient registry or patients who have redeemed ≥2 prescriptions for relevant cough-medication within a 90-day harvest in the Prescription registry from 2008 to 2017. To validate this study’s chosen proxy on chronic cough an analysis of the Patient registry sub-population with a contact of ≥8 weeks and then final diagnosis code DR05/DR059 was also performed. The population was divided into UCC and RCC.

**Results:**

Of the 104,216 patients from the Prescription registry, 52,727 were classified as having UCC and 51,489 were classified with RCC. From the Patient registry 34,260 were included, of whom 12,278 had UCC and 21,982 had RCC. Cough were frequently found among females (*p* < 0.0001). Both genders were around 2 years older in RCC than UCC (*p* < 0.0001) Spirometry was performed in 69 and 57%, X-ray in 73 and 58% and asthma challenge test performed in 13 and 5% (UCC and RCC, respectively, p < 0.0001). The frequency of co-morbidities such as heart failure, rheumatologic disease, pulmonary embolism, and diabetes was < 10%.

**Conclusion:**

Many patients suffer from chronic cough or cough requiring medications, with or without co-morbidity; frequently found among menopausal women. Most patients had a substantial work-up performed. The high frequency and the resources consuming work-up program call for systematic coding of disease, systematic patient evaluation and more specific treatment options. The study was approved (ID: no. P-2019-191).

## Background

Cough is one of the most common reasons for contact with the primary health sector [1]. Cough may be present as an acute, subacute or a chronic condition, where the latter may be very disturbing for the patients and difficult to treat for the medical staff.

Coughing is a normal physiological process; it is a protective reflex that clears debris and secretions from the airways. The cough reflex consists of an afferent sensory limb and the central processing centre, activating the efferent limb. The afferent nerves involved are the vagal nerve and it’s sensory branches. These cough important sensory nerves are found from the pharynx to the terminal bronchioles; most are located in the larynx, carina and bifurcation of the larger bronchi [2]. The signal that irritants are present in the bronchial system is mediated to the cough centre in the brainstem. The efferent pathways are then mediated back to the respiratory tract through the vagal, phrenic and spinal motor nerves [3]. This causes activity in the respiratory muscles, with closure of the vocal cords and activation of the bronchial smooth muscles. This mechanism is present in cough as a protective mechanism, in cough as a symptom of underlying conditions, and as a reflex which has become dysregulated. Chronic cough is often, but not always, associated with different co-morbidities both in the respiratory area, but also outside the thorax [4]. The most common underlying conditions in patients with chronic cough are upper airway cough syndrome, chronic eosinophilic bronchitis, asthma and gastro-esophageal reflux disease (GERD) [5]. Coughing is a well-known side effect of treatment with ACE-inhibitors as up to 20% of the patients treated with an ACE-inhibitor reported coughing as a side effect, which can lead to treatment termination [6].

The true prevalence of chronic cough in the clinical setting, either as an unexplained symptom or as a refractory symptom in relation to a co-morbidity, is not known, since cough seldom is registered in a patient registry as a secondary diagnosis or a co-morbidity, when other diseases are present [7].

Cough in population studies is often registered as part of the questionnaire based respiratory symptoms of chronic bronchitis [8], where cough might be the only symptom present. In a Danish population survey, self-reported chronic cough has been found to have a prevalence of 4% among never-smokers and 8% in a subgroup of current smokers [9]. The frequency of cough in a European study was estimated to be 12.7%, indicating geographic differences but possibly also a difference in methodology of recording and definition of cough) [10].

In the present paper, chronic cough is defined as coughing for more than 8 weeks. Chronic cough potentially related to other co-morbidities is defined as Refractory chronic cough (RCC), whereas chronic cough without any other diseases is defined as Unexplained chronic cough (UCC) [11]. Yet, knowledge about the exact awareness of cough, use of cough treatment, co-morbidity treatment, and evaluation schemes of cough among adults is limited. In this registry study, we aim to examine the frequency of chronic cough, co-morbidities, treatment, factor of importance and demographics in a Nationwide study.

## Material and methods

### Design

This is a descriptive retrospective observational database-registry study, using data from the Danish National Patient Registry (Patient Registry) and The Danish National Prescription Registry (Prescription Registry). All inhabitants in Denmark are assigned a unique social security number (CPR number) at birth or immigration. It is not possible to select Danes born in Denmark and immigrants, they all have the same Unique social number. This unique social security number is used in all public health registries such as the Patient Registry and the Prescription Registry and for registration of all contacts with the health system, i.e. general practitioners (GP), specialist care out-side hospital, private hospitals as well as examinations and treatment performed in hospital settings. The importance of this, is that we gain data from both primary and secondary health care. The Danish Data Protection Agency have approved the use of the Danish National Health Data for use in this study, in a anonymized form. The use of anonymized registry data does not require ethical approval in Denmark. The study was approved by the Danish Data Protection Agency (ID: no. P-2019-191).

## Material

### Study population

Inclusion criteria were patients ≥18 of age and below 90 with at least one final ICD-10 Patient registry diagnosis code of DR05/DR059 (cough/cough with no further specification) in the Danish National Patient Registry – and/or – having redeemed ≥2 prescriptions for relevant cough-medication within a 90-day period in the Danish National Prescription Registry in the 10-year period from January 1st 2008 to December 31st 2017. Two or more prescriptions within 90-days support that the need of medication is a chronic use. ATC Code: Anatomical Therapeutic Chemical Classification System and International Classification of Diseases (ICD) codes of version 10 was used (ICD-10).

The study consists of three populations from the two national validated databases:

#### Prescription registry population – group 1 (Fig. 1 a)

Included in the prescription registry population are patients who have redeemed ≥2 prescription medications within 90 days to treat cough (Prescription registry *ATC code R05*) and including ATC codes for Codeine (R05DA04), Noscapine (R05DA07), Dextromethorphan (R05DA09), Pectyl (R05FA02), Opium drops (*A07DA02*) and all mucolytics (R05CB). Furthermore, for the identification of co-morbidities possibly relating to cough, a search for upper and lower respiratory drugs (Prescription registry ATC codes R03 and R06) and reflux (ATC Patient registry: A02) were identified. The Prescription registry population represents primary care praxis since most cough related medications will be prescribed by general practitioners.

**Fig. 1:**
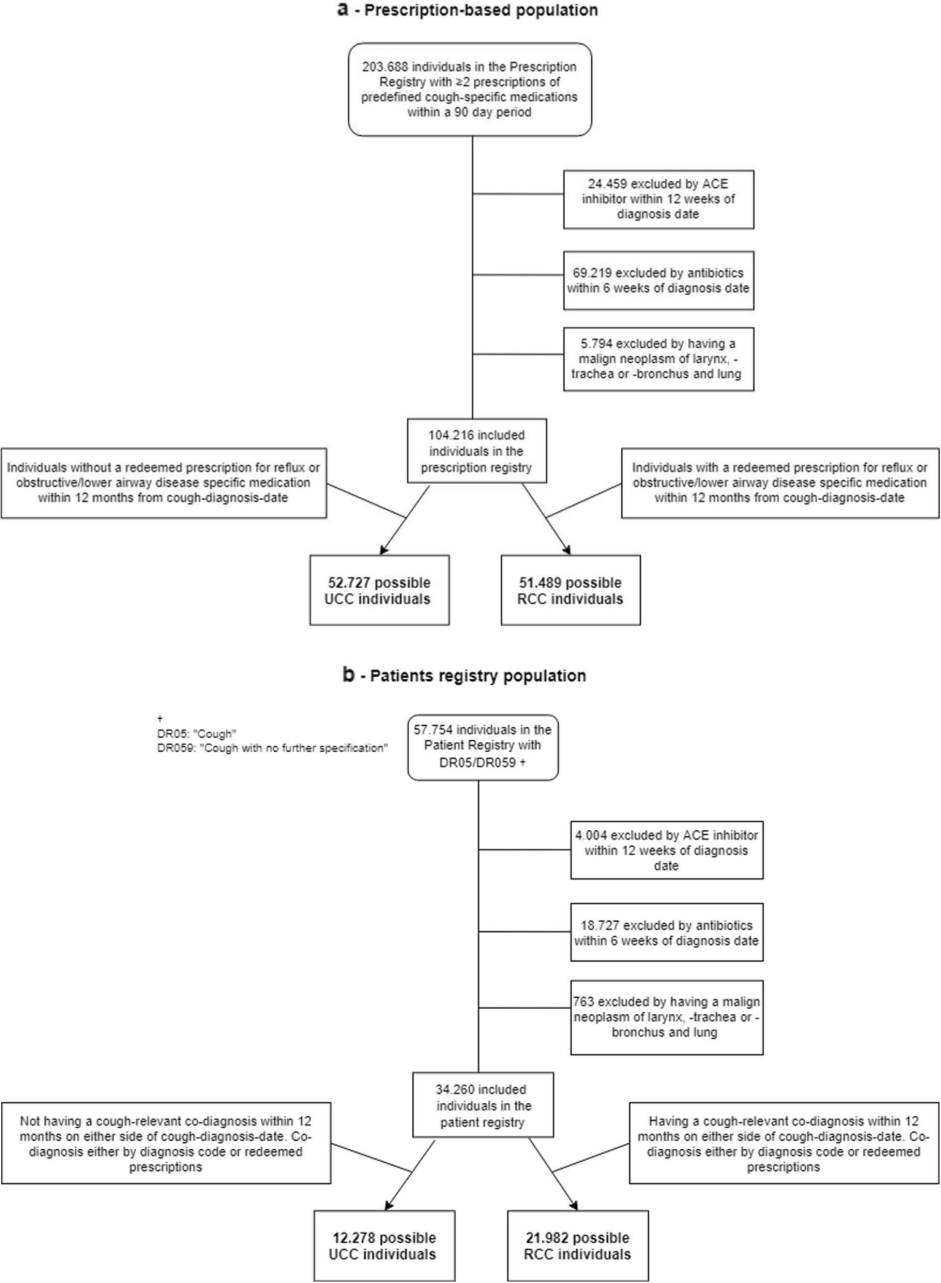
**a** Patient-flow in population-based prescription registry. **b** Patient-flow in patients-based registry

#### Patient registry population - group 2 (Fig. 1 b)

Included in the population based on diagnosis from the patient registry are patients who have been diagnosed with the cough-specific diagnosis (DR05/059) in the National Danish Patient Registry.

The identification of co-morbidities possibly relating to cough were done by searching the diagnosis registry for chronic lower respiratory disease coded with DJ40–46, including COPD (DJ44) and asthma (DJ45). Acute lower respiratory diseases (ICD-10 Patient registry of DJ20–22), upper respiratory illnesses (ICD-10: DJ30–39) and lastly, GERD was based on ICD-10 code DK21, and psychogenic cough on ICD-10 code DF453. Non-respiratory illness which might lead to cough, such as heart failure (DI24–25), different types of arthritis (DM051–52, and DM060–69), pulmonary embolism (DI26) and diabetes (DE10–11) were examined as well. The Patient registry population represents secondary health care from hospital/specialist setting.

#### Combined prescription- and patient registry population – group 3

A merged population with patients present in both the Prescription Registry and Patient Registry databases using the above inclusion criteria which represents the group of patients who have been in contact with both primary and secondary health care and furthermore, when prescription of anti-cough medication has been both prescribed and collected.

**Exclusion criteria** was the use of ACE-inhibitors (C09AA) within 12 weeks of diagnosis date, antibiotics (J01) within 6 weeks of diagnosis date and any malignant respiratory disease in the larynx (ICD-10 C32), tracheus (C33) or bronchus/lung (C34).

### Registries used

The Danish National Patient Registry records all ICD-10 diagnoses given within the Danish healthcare system which enables tracking of all patient contacts [12]. There is a strong economic incentive for the hospital departments/specialists to use the system, since the Danish National Patient Registry uses the ICD-10 and testing codes for reimbursement [12].

The Danish National Prescription Registry records all specific prescriptions’ ATC codes redeemed from Danish pharmacies also using the unique social security number. Over the counter medications are not recorded in the prescription registry and are therefore not possible to include in this study.

The Prescription registry population (group 1) is allocated from the first event of ≥2 prescriptions redeemed within 90 days in the 10-year study period. The 90 days were selected, due to the content of the package of cough medication and the devices in respiratory medicine. Tablets towards cough are most often prescribed with 40 tablets per package, and 2 prescriptions are needed within less than 90 days. In case of 3 tablets per day, a package of 100 tablets would last 33 days and in case of 2 tablets daily the amount will last 50 days. Mixture of cough medication would be prescribed in 200 mL per bottle, with the lowest doses of 5 mL, lasting 40 days. Respiratory devices containing 30 doses for treatment once daily and 60 doses for treatment twice daily, i.e. one device per month, some packages might include 3 devices, which cover treatment for 90 days, but not 91 days, therefore number 2 prescriptions would be needed within 90 days, for continuation of treatment and this cut-off was selected in the current survey. The Patient registry population (group 2) is based by first occurrence of a final ICD-10 codes from a contact in the patient registry for cough-diagnoses (DR05 and DR059) and therefore patients only occur once in either population.

#### Cough specification

Chronic cough is defined as cough > 8 weeks [11]. In this study the definition used for chronic cough is ≥2 redeemed prescriptions within 90 days or having a contact with the healthcare system where final diagnosis of the contact being ICD-10 code DR05/DR059. A final diagnosis code of cough from a health care contact can be used as a proxy for chronic cough since the patient will be referred by a general practitioner after a longer period of coughing before the hospital contact. The other reason is the economic incentive from the hospital/specialist to use a ‘real’ ICD-10 diagnosis code for reimbursement and only use cough if no other diagnosis is present. To validate this study’s chosen proxy on chronic cough an analysis of the Patient registry sub-population with a contact of ≥8 weeks and then final diagnosis code DR05/DR059 was also performed.

The included populations were identified as having either Possible Unexplained Chronic Cough (UCC) or Possible Refractory Chronic Cough (RCC). Possible UCC being defined as not having a cough-relevant co-diagnosis within 12 months before and after the point at which the diagnosis date was registered and possible RCC being defined as having a cough-relevant co-diagnosis within 12 months on either side of cough diagnosis-date. For the Prescription registry population co-morbidities were identified through other prescriptions if they did not also have a contact in the Patient registry.

### Outcomes of the study

The outcomes of the study were pre-defined and were as follows:

#### Primary outcome

Baseline characteristics and prevalence of individuals with possible UCC– including demographic composition and characteristics as well as relevant examinations in relation to diagnosis.

#### Secondary outcomes

Baseline characteristics of individuals with possible RCC – including demographic composition and characteristics, cough-related comorbidities, as well as relevant concomitant medications and relevant examinations in relation to diagnosis.

**Demographic data** such as age, gender, and living region in Denmark (Capital city area, urban area, rural area) is registered in the Patient registry, whereas hospital-based prescription and hospital-based treatments such as biological drugs is not accessible through the Prescription Registry and is therefore not reported if the patients are only registered in the Prescription Registry. The cough-relevant medication redeemed at the pharmacies for inclusion in the prescription-based population is reported (see below) [1]. Respiratory medication R03, antitussive and cold medication (R05), antihistamine (R06), and lastly, opium-drops (A07/DAO2).

#### Examinations and tests

For the Patient registry population a search for relevant examinations related to cough, such as lung function testing, asthma provocation, chest x-ray, CT-scan of thorax, HRCT of the lungs, gastroscopy, laryngoscopy, and bronchoscopy was performed (360 days on each side of diagnosis-date) to describe the relevant examinations in connection with cough diagnosis-date. It is not possible to perform a search for relevant examinations for the Prescription registry population; therefore this is not reported. There was no access to patient records and blood tests.

### Statistics

Data analysis was performed using SAS for Windows (SAS, Cary, NY, US) version 77.1. Categorical variables are described as absolute numbers as well as percentage where possible. All data generated or analysed during this study are included in this published article. The analysis for demographic data included all inhabitants from Denmark, based on prescription lists and ICD-10 Patient registry’s, co-diagnosis, co-medications, and relevant testing as well as examinations were performed for the population of the primary (UCC) and secondary outcome (RCC). A merge of the Prescription registry population and the Patient registry population within both UCC and RCC was performed. Statistical analysis (Chi square) has been performed between UCC and RCC, and a *p* value of < 0.003 are regarded as significant, corrected for multiple analysis (*n* = 15).

## Results

Over a period of 10 years (2008–2017), 203,688 patients had redeemed ≥2 relevant prescriptions within 90 days, of which 99,472 were ineligible and 104,216 were included in the Prescription registry population (group 1) (Fig. 1 a). During the same period 57,754 patients were given diagnosis of cough in the Patient registry, of whom 23,494 were ineligible and 34,260 were included (group 2) (Fig. 1 b). 4004 (6.9%) patients were excluded due to ACE inhibitor initiated less than 12 weeks prior to the diagnosis of cough. In the combined Prescription and Patient registry population 11,209 patients were registered to have both ≥2 redeemed prescriptions as well as the diagnosis cough. Of these 8024 were ineligible and 3185 were included (group 3).

All patients included are sorted in Table 1 using the definitions for UCC (no relevant co-morbidity ±12 months from diagnosis) and RCC (cough-relevant co-morbidity related to diagnosis) and divided into three groups (Prescription registry, Patient registry and the combination of the two). Of the 104,216 patients from the Prescription registry group, 52,727 were classified as having UCC (primary outcome) and 51,489 were classified with RCC (secondary outcome). Of the 32,260 patients from the Patient registry, 12,278 were classified as having UCC (primary outcome) and 21,982 were classified as having RCC (Table 1). There were 3185 patients present in both the Prescription registry and the Patient registry, where 529 were classified as having UCC and 2656 as having RCC.

**Table 1:**
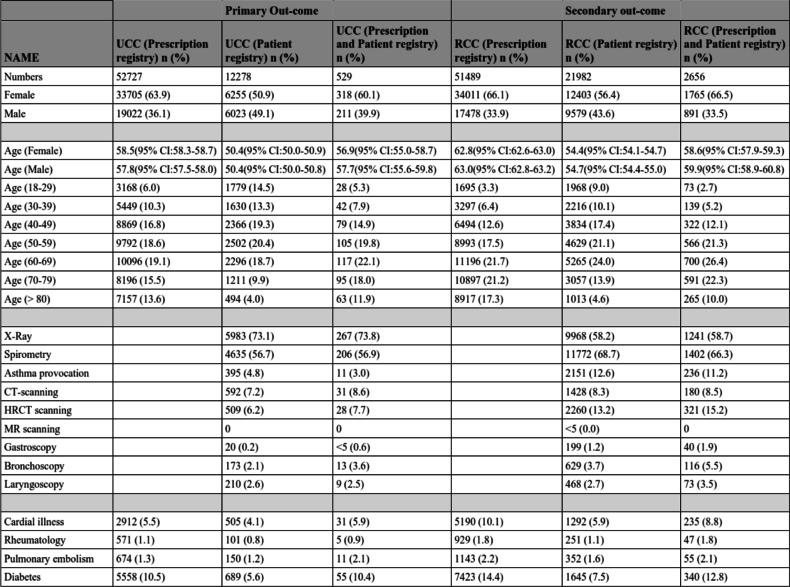
Demographic data 2008–2017 (10 years)

More females than males were diagnosed with cough in the group of possible UCC (Table 1, *p* < 0.0001), and an even more pronounced skewness towards females was observed in the group with possible RCC. The age varied between 50 and 66 years, with no age differences between females and males in all groups (Table 1). The mean age of females with UCC 56.88 and RCC 58.60 (*p* < 0.0001), and among males 57.70 and RCC 59.86 (*p* < 0.0001). Furthermore, the data have been grouped into age-related intervals. There was a skewed distribution of patients with chronic cough (data not shown), point prevalence with an overweight among the bigger cities, however an exact calculation is not possible due to the move of patients around in the area.

In the entire population, those selected through the Patient registry with the diagnose of cough, where different testing possibilities was coded as well, the most frequently performed examination was spirometry (*n* = 16,407) followed by X-ray (*n* = 15,951), CT-thorax (*n* = 2020) and HRCT of the lungs (*n* = 2769) and lastly bronchial provocation (*n* = 2546). When dividing the total number of examined participants into UCC and RCC cohorts, 57% had spirometry, 73% X-ray, and 5% had bronchial challenge performed among those with UCC (Table 1) and 69% had spirometry, 58% had X-ray, and 13% had bronchial challenge test performed among RCC (Table 1).

The number of patients with UCC having X-ray performed was higher than RCC (*p* < 0.0001), whereas all other tests (FEV1, asthma test, CT, HRCT; gastroscopy and bronchoscopy (Table 2)) was more frequently performed in patients with RCC than UCC. Whereas no differences was found between the number of patients who had laryngoscopy performed (Table 2).

**Table 2:**
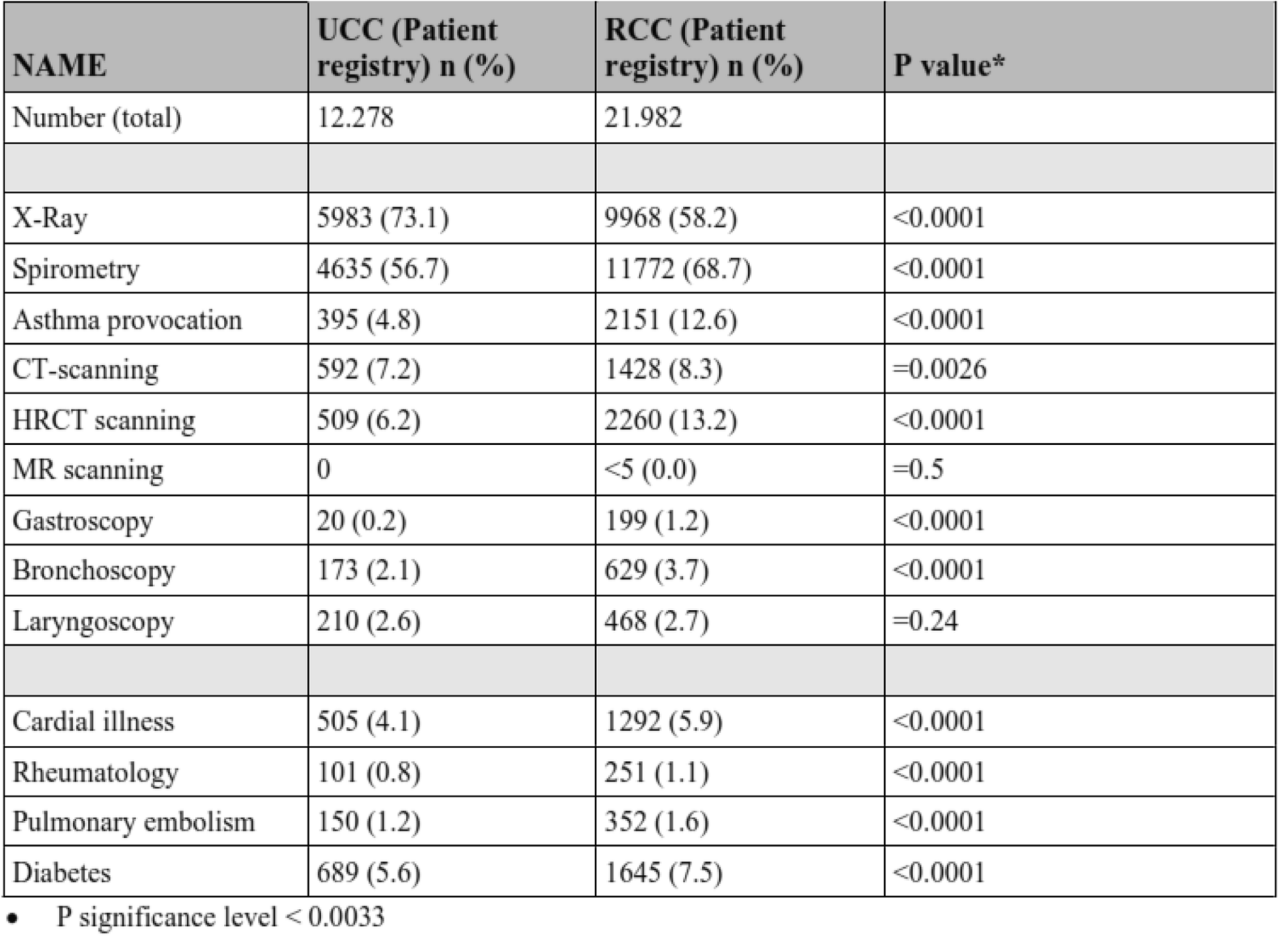
Analysis of significant differences between UCC and RCC and both different tests performed and co-morbidities (% of column participants)

Other co-morbidities such as heart failure, rheumatologic disease, pulmonary embolism, and diabetes are also shown in Table 1, and the frequency is low and with similar distributions in both UCC and RCC. Similar, cardiac co-morbidities were less frequent in the group of UCC (between 4 to 6%), compared to RCC (between 6 to10%), whereas no such differences were found among the other selected co-morbidities (Table 2, *p* < 0.0001).

Lastly, an extra analysis has been performed in patients who have had attachment to the department for 8 weeks or more followed by a diagnosis of cough or a prescription of the selected medication. This included patients diagnosed as UCC and RCC, 2442 and 5204, respectively, and when including the prescription and the diagnosis, it was 114 and 637 patients, respectively. The distribution of all the variable included, was similar as in the main group, thus fewer (data not shown).

## Discussion

In this nationwide study and over a period of 10 years from 2008 and 2017, we have shown that cough encounters are numerous and both patients with unexplained chronic cough (UCC) and refractory chronic cough (RCC) are frequently found. When using the Patient registry to identify the frequency of UCC versus RCC, it is found that there are twice as many patients with RCC, which correlates well with other studies on the ratio on UCC vs RCC found in a specialist setting [10]. However, when examining the frequency of possible chronic cough based on the use of cough medications through the Prescription registry, the ratio between UCC and RCC were similar in the two groups, the latter of which might be explained by the reduced likelihood of coding for cough in hospital setting due to reimbursement policy or that the captured number of cough patients by the prescription list is too difficult.

In this study we found an overweight of females (62%) and in general the patients were above the age of 50 years, which is similar to findings in other studies reporting demographics of patients with cough [13–16]. Patients with RCC were a little older than patients with UCC, independent of gender. Importantly, no differences in age and gender were found based on the selection criteria being by the Prescription or Patient registry. Indicating that the selection is unbiased and independent of the methods used.

This is to our knowledge one among few studies, examining chronic cough from both primary care using the Prescription registry, and secondary care using the Nationwide ICD-10 Patient registry. The Prescription registry’s list of cough relevant medications leads to a set of data with more than 100.000 patients over 10 years. However, this large group only represents around 3% of the adult Danish population and when analyzing the group of patients coded with cough using the patient registry it only represents 0.9% of the Danish population. These frequencies of cough are low compared with other studies [14]. In a population study, it was found that 4% suffered of self-reported chronic cough among the entire Danish population, thus only 3% suffered of chronic cough among never smokers [9]. The frequency of chronic cough was higher in populations of COPD patients (10%) [17], as well as patients with asthma (8%) [18]. These frequencies are thus relative low, as the frequency of cough are found to vary between 4 to 33% [9, 19]. This indicates that the current study might underestimate the frequency of cough in the society when using registries to identify patients, and a possible explanation being that cough is often viewed as a symptom rather than a medical condition and is used accordingly less frequent than ‘proper’ diagnoses such as pharyngitis, possible asthma, reflux, but not COPD, as that would need low level of lung function. In a survey by Weiner et al. [15], they demonstrated a similar low frequency of coded chronic cough, while the frequency of cough described in electronic patients files and data capturing was substantially higher, indicating, that many patients are complaining of cough, but neither a coding of the cough diagnose, due to lack of specific ICD.-10 codes, or prescription of relevant medication has been performed. These findings of diminished registration of cough, most likely both in our study and in other surveys, are relevant in the future, when further development in the area might come. In the future this might change as, we in Denmark have developed an formal ICD-10 code called 05.97. Studies of chronic cough have shown low level of quality of life in those with the highest level of cough complains, indicated by a high Leicester Cough Questionnaire score and a low Cough Quality of Life Questionnaire score [CQLQ]), with a correlation coefficient of minus 0.80 [13].

The merged Prescription and Patient registry population with UCC showed a relatively small number of patients within the UCC-group whereas those with RCC were relatively more frequent, indicating that a substantial group of patients suffers from chronic cough on top of their underlying main disease. The merged group might be a more trustfully group of patients, as they have both the symptoms and the prescription of relevant medication and has been seen in both primary and secondary care. The reason for the imbalance between the patients prescribed relevant medication and not given the diagnosis of cough in hospital setting, might be a lack of referral to hospital or that patients are given another diagnosis than cough due to a lack of proper ICD-10 code. The study set-up does not make any possibility of explanation. In patients with persistent cough despite treatment for an underlying cause (ie the RCC-population) the persistent cough might be viewed and treated as an uncontrolled disease with larger doses asthma- or reflux medication, whereas persistent cough of unknown cause is more difficult to evaluate and treat. New approaches with other treatment options focusing more on the coughing reflex might alter this phenomenon.

The use of examinations and tests performed in both the UCC and RCC groups are numerous and pointing towards a wide variety of specialties such as pulmonology, rhinology, gastroenterology, and cancer diagnostics. Asthma provocation test was performed in 4.8% of UCC versus 12.6% in RCC. The most serious cause of chronic cough is lung cancer, and therefore all with cough should have had an X-ray performed, but the findings in the study was only 73% in the population with UCC. These findings are higher than in similar studies by Zeiger et al. [14], whom found that 62% have had an X-ray taken, where fewer had spirometry performed. Furthermore, since the most frequent cause of chronic cough is COPD or asthma, spirometry should also have been performed in all patients and data show the frequency of spirometry performed was even lower than performed X-rays. (Table 1). These findings suggest that although guidelines exists they might not always be followed when examining a patient with cough and chronic cough [19], and this paper including the work-out of patients suffering of possible chronic cough suggest the need for systematic evaluation. There are several different clinical pathways, when evaluating chronic cough and symptoms of cough calls for collaboration between various specialties, as e.g. upper airway illness with post-nasal drip and gastro-esophageal reflux also leads to cough [20–22]. A multidisciplinary approach might reduce the time spent for both patients and hospital and this is important on a national level since currently differences in culture exists between urban and rural areas. The skewness of cough towards inner city cough with higher frequency (data not shown), might also be related to environmental factors, such as pollution which is known to increase the level of symptoms [23].

### Strengths and limitations

One of the strengths of this study is that both the Prescription and Patient registries are validated on a national level, giving them a high level of credibility and there is a strong economic incentive to use the system, since the ICD-10 coding, given at the final visit, leads to reimbursement and payment of the hospital department or specialist practice. In the country involved in this study, we have one number each, same number use in Medicare, social welfare, education, medication and use of hospital facilities. All Medicare is free of charge for all inhabitants, independent of income or not. This support, that the data could be generalized, although it is a one nation study.

The weakness in this registry-based study is, that it is not possible to identify and exclude smokers or obese patients, which are known reasons for cough and tobacco cessation has been shown to reduce cough substantially [9, 24]. It is a limitation in this study that surrogate measures are used to define chronic cough, both from the Prescription database and the Patient registry, even though measures were taken to eliminate reasons for acute cough through exclusion of antibiotics, cough due to ACE-inhibitors and diagnosed cancers as well as not having the exact time frame of the cough prior to final diagnosis in the Patient registry, even though in the Danish clinical setting the final diagnosis can be defined as a contact lasting for ≥8 weeks. One could argue that patients treated with ACE develop chronic cough, whereas only 7% had the diagnosis of cough less than 12 weeks before and were deleted from the cohort. From a clinical point of view, patients with cough, and treatment with ACE, are discontinued with the treatment to test the relationship between the two. As it is a register-based study, we cannot examine this, which might be a limitation of the study. Furthermore, another issue could be Codeine selected as a cough suppressant drug, which also have pain killer effect. We believe that this is only of minor importance, as the doses used in the analysis are equivalent to the prescribed doses for cough treatment. The number of patients, with the strengthen criteria, attachment to the department of 8 weeks and more, followed by a diagnosis of cough were reduced in number of included patients, but the distribution was similar. We therefore consider the main group as the final diagnosis and analysis.

In the Patient registry the diagnosis of cough is dependent on physician performing the right coding, and since cough being a symptom diagnosis and not a distinct medical condition it could be skewed due to the re-imbursement policy, but it will not be a source of bias to the data reported but only result in underreporting. In the future, it would be helpful to have specific disease code for cough and not only a code for symptoms such as cough, as a large evaluation program are used, with high cost and many visits, which should be relevantly re-imbursed, and furthermore, when new treatment possibility develops, it might be of importance to follow the flow of patients ensure the quality of treatment.

## Conclusion

Many patients suffer from chronic cough or cough requiring medications, with or without co-morbidity; frequently found among menopausal women. Most patients had a substantial work-up performed. The high frequency and the resources consuming work-up program call for systematic coding of disease, systematic patient evaluation and more specific treatment options. This calls for a multi-specialty approach at specialized centers and illustrates the need for future therapeutic options. The new ICD-10 code covering cough might increase the validity of these studies, which will increase the possibility for further treatment of the patients and even better research.

## Data Availability

The data is on the National database, we have performed analysis and developed a syntax file so we could do the same analysis again. No one is allowed to take data out of the National database, but you need specific allowance to analyze the data (ID: no. P-2019-191), which we had.

## Abbreviations

RCC: Refractory chronic cough
UCC: Unexplained chronic cough
GERD: Gastro-esophageal reflux disease
GP: General practitioners
CPR number: Social security number
ATC Code: Anatomical Therapeutic Chemical Classification System
ICD-10: International Classification of Diseases (ICD) codes of version 10.
